# IFI35, mir-99a and HCV Genotype to Predict Sustained Virological Response to Pegylated-Interferon Plus Ribavirin in Chronic Hepatitis C

**DOI:** 10.1371/journal.pone.0121395

**Published:** 2015-04-06

**Authors:** Emilie Estrabaud, Kevin Appourchaux, Ivan Bièche, Fabrice Carrat, Martine Lapalus, Olivier Lada, Michelle Martinot-Peignoux, Nathalie Boyer, Patrick Marcellin, Michel Vidaud, Tarik Asselah

**Affiliations:** 1 INSERM, UMR1149, Team «Viral hepatitis », Centre de Recherche sur l’inflammation, BP 416, Paris, France; 2 Université Denis Diderot Paris 7, site Bichat, BP 416, Paris, France; 3 Service d’Hépatologie, PMAD Hôpital Beaujon, 100 Bd du Général Leclerc, Clichy la Garenne, Clichy, France; 4 Laboratory of Excellence Labex INFLAMEX, PRES Paris Sorbonne Cité, France; 5 UMR745 INSERM, Université Paris Descartes, Sorbonne Paris Cité, Faculté des Sciences Pharmaceutiques et Biologiques, Paris, France; 6 Unité de santé publique, Hôpital Saint-Antoine, Assistance Publique Hôpitaux de Paris and UMR-S 707, UPMC Université Paris 06 & INSERM, Paris, France; National Taiwan University Hospital, TAIWAN

## Abstract

Although, the treatment of chronic hepatitis C (CHC) greatly improved with the use of direct antiviral agents, pegylated-interferon (PEG-IFN) plus ribavirin remains an option for many patients, worldwide. The intra-hepatic level of expression of interferon stimulated genes (ISGs) and the rs12979860 CC genotype located within IFNL3 have been associated with sustained virological response (SVR), in patients with CHC. The aim of the study was to identify micro-RNAs associated with SVR and to build an accurate signature to predict SVR. Pre-treatment liver biopsies from 111 patients, treated with PEG-IFN plus ribavirin, were studied. Fifty-seven patients had SVR, 36 non-response (NR) and 18 relapse (RR). The expression of 851 human miRNAs and 30 selected mRNAs, including ISGs, was assessed by RT-qPCR. In the first group of patients (screen), 20 miRNAs out of the 851 studied were deregulated between NRs and SVRs. From the 4 miRNAs validated (mir-23a, mir-181a*, mir-217 and mir-99a), in the second group of patients (validation), 3 (mir-23a, mir-181a* and mir-99a) were down-regulated in NRs as compared to SVRs. The ISGs, studied, were accumulated in SVRs and IFNL3 rs12979860 CT/TT carriers compared respectively to NRs and CC carriers. Combining, clinical data together with the expression of selected genes and micro-RNAs, we identified a signature (*IFI35*, mir-99a and HCV genotype) to predict SVR (AUC:0.876) with a positive predictive value of 86.54% with high sensibility (80%) and specificity (80.4%). This signature may help to characterize patients with low chance to respond to PEG-IFN/ribavirin and to elucidate mechanisms of NR.

## Introduction

Hepatitis C virus (HCV) is a major cause of chronic liver disease, with more than 170 million infected individuals, worldwide [1,2].

The treatment of chronic hepatitis C (CHC) has greatly improved, with the development of direct antiviral agents (DAAs), reviewed in [3,4]. Whereas interferon-free combinations are very effective, pegylated-interferon (PEG-IFN) remains an option for a large number of patients worldwide mainly because of cost issues and access to treatment. Therefore, the prediction of SVR to IFN based therapy is still highly important to identify (i) patients with high chance to cure and therefore candidates for this therapy and (ii) those with low chance to respond to PEG-IFN plus ribavirin, candidates for IFN free therapies. Moreover, the prediction of non-response may help to elucidate its molecular mechanisms. The SVR rate is highly associated with viral factors; patients with genotype 1 and 4 show significantly lower response rate than patients with genotype 2 and 3. Different liver gene expression profiles have been associated with non-response, reviewed in [5]. Interferon stimulated genes (ISGs), have been reported over-expressed, at baseline, in patients with non-response (NR) compared to sustained virological responders (SVR) [6–9]. It has been suggested that in patients with high expression of ISGs, at baseline, the addition of exogenous interferon was not able to activate ISGs expression further [7,9].

Single nucleotide polymorphisms (SNPs), in particular rs12979860, located upstream *IFNL3*, were strongly associated with SVR [10,11]. Patients, carrying the favourable genotype rs12979860 CC, have higher chance to develop a SVR than patients with CT and TT genotypes.

Micro-RNAs (miRNAs) are small non-coding RNAs controlling the expression of up to 60% of total mRNAs and therefore are involved in the regulation of major cellular pathways [12,13].

Both *in vitro* and *in vivo* models showed variation of cellular miRNAs expression upon HCV infection [14–16]. Moreover, IFNα/β up-regulates several cellular miRNAs (mir-196, mir-296, mir-351, mir-431, mir-1, mir-30 and mir-128 and mir-448) with putative recognition sites within HCV genome [17]. Mir-196, mir-296, mir-351, mir-431 and mir-448 were indeed able to substantially attenuate viral replication [17]. Mir-122 is highly expressed in the liver; its over-expression stimulates HCV replication, *in vitro* [18]. The use of anti mir-122 (SPC3649, miravirsen) inhibits viral replication both in animal model and in HCV infected patients [19–21]. A reduction of mir-122 expression has been reported, at baseline, in patients with primary non-response as compared to patients with early virological response [22]. In a previous work, we showed that mir-122 expression was decreased in patients with IFNL3 CT/TT genotypes and that this association was stronger than the one between mir-122 and the response to the treatment [23].

We aimed to identify a specific miRNAs expression profile associated with SVRs and NRs, in patients with chronic hepatitis C. We studied the expression of liver miRNAs in two independent groups of patients (screen and validation groups). We investigated the expression of validated miRNAS, in serum samples. We built a predictive signature of SVR taking into account clinical and biological data, *IFNL3* rs12979860 genotype and intra-hepatic mRNAs and miRNAs expression.

## Patients and Methods

### Characteristics of patients

One hundred eleven patients with CHC, followed at Beaujon Hospital, were consecutively included in the study. Pre-treatment percutaneous liver biopsies were collected. The study conformed to the ethical guidelines of the 1975 Declaration of Helsinki. All participants provide their written informed consent to participate in this study.

The patients met the following criteria:

- an established diagnosis of CHC
- the absence of other cause of chronic liver disease and viral co-infection
- the patients received a complete treatment of either PEG-IFNα-2b (MSD) at a dose of 1.5 μg/kg/week and weight-based ribavirin at a dose of 800–1200 mg/day, or PEG-IFNα-2a at a dose of 180 μg/week (Roche) and weight-based ribavirin 1000–1200 mg/day. Duration of treatment was 48 weeks for HCV genotype 1 and 4 and 24 weeks for genotypes 2 and 3. For HCV genotype 1 and 4, If HCV-RNA was detectable at 24 weeks of therapy, treatment was stopped (non response).
- the patients had an adequate follow-up: detection of serum HCV RNA by RT-PCR was performed at week 12, week 24, at the end of treatment and and 24 weeks after the end of treatment. SVR was defined as undetectable HCV RNA 24 weeks after completion of treatment, while NR was defined as detectable serum HCV RNA at the end of the treatment [24,25]. Responder-relapsers (RR) was defined as the reappearance of detectable serum HCV RNA within 24 weeks, after treatment cessation in patients with no detectable serum HCV RNA at the end of treatment [24,25]. All the patients included in this study had an excellent compliance.

A total of 111 patients were included in two independent groups (Table 1): the screen group (n = 28; 14 NRs and 14 SVRs) and the validation group (n = 83; 42 SVRs, 22 NRs and 18 RRs). The differential expression of the 851 human miRNAs in NRs and SVRs was assessed in the screen group, therefore the same number of patients with SVR and NR were included. Liver biopsies from the validation group (n = 83; 42 SVRs, 22 NRs and 18 RRs) were used in an independent set of experiments, to confirm the results. We enlarged the population with SVRs, NRs and RRs, in accordance with real life data. The expression of the validated mRNAS was assessed in available serum samples from 68 patients of our cohort. The 20 miRNAs deregulated in the screen group (showing at least a 2-fold difference between NRs and SVRs) were studied independently in the validation group.

**Table 1:** Characteristics of the patients.

**Variable**		**Screen**	**Validation**	**Total**	**SVR/NR Pvalues** ^b^
**n**		28	83	111	
**Gender: male (%) / female (%)**		14(50) / 14 (50)	56 (67) /27 (33)	70 (63) / 41 (37)	0.1315
**Age [mean ± SD (range)]**		50.5 ± 7.5 (34–68)	46.6 ± 9.1 (28–73)	47.1 ± 8.9 (28–73)	
**BMI, kg.m-2 [mean ± SD (range)]**		25.08 ± 3.64 (19.37–33.05)	25.49 ± 4.21 (17.64–37.83)	25.45 ± 4.19 (17.64–37.83)	0.0967
**Alanine minotransferase (ALT) IU/L [mean ± SD (range)]**		103.0 ± 97.9 (20–352)	107.1 ± 67.1 (18–459)	105.1 ± 72.5 (18–459)	0.5508
**Aspartate aminotransferase (AST) IU/L [mean ± SD (range)]**		65.0 ± 57.6 (25–233)	63.87 ± 35.49 (21–323)	64.07 ± 34.80 (21–323)	0.1565
**Gamma-glutamyltransferase (GGT) IU/L [mean ± SD (range)]**		64.73 ± 90.78 (3–315)	90.31 ± 86.27 (7–411)	84.98 ± 87.20 (4–411)	0.0040
**Total-cholesterol mmol/L [mean ± SD (range)]**		4.80 ± 0.77 (3.43–6.49)	4.70 ± 0.92 (3.11–7.36)	4.73 ± 0.87 (3.11–7.36)	0.0829
**Glycemia mmol/L [mean ± SD (range)]**		5.0 ± 0.63 (3.2–6.3)	5.40 ± 1.50 (3.14–13.6)	5.2 ± 1.31 (3.2–13.6)	0.9353
**Viral loads: copies per mL [median (range)]**		4.63.106 (1.4.104–2.73.107)	1.87.106 (3.103–3.06.107)	2.97.106 (3.103–3.06.107)	0.0032
**HCV genotypes, n(%)**	1	19 (68)	38 (46)	57 (51.2)	1 vs. 2,3,5,6 0.0029
	2	3 (10.5)	9 (10.5)	12 (10.8)	
	3	1 (3.5)	13 (15.5)	14 (13)	
	4	4 (14.5)	22 (26.5)	26 (23.2)	
	5	1 (3.5)	1 (1.5)	1 (0.9)	
	6		1 (1.5)	1 (0.9)	
**IFNL3 rs12979860 polymorphism, n(%)**	C/C	11 (39.3)	20 (24.1)	31 (28)	CT vs. CC 0.2634 TT vs. CC 0.0062
	C/T	7 (25)	33 (39.8)	40 (36)	
	T/T	8 (28.6)	19 (22.9)	27 (24.3)	
**Fibrosis Score (Metavir), n(%)**	1	11 (39.2)	23 (27.7)	34 (30.6)	0.5835
	2	16 (57.2)	24 (28.9)	40 (36.1)	
	3	1 (3.6)	17 (20.5)	18 (16.2)	
	4		17 (20.5)	17 (15.3)	
	unknown		2 (2.4)	2 (1.8)	
**Treatment response, n(%)** ^**a**^	SVR	14 (50)	43 (52)	57 (51.2)	
	NR	14 (50)	22 (26.5)	36 (32.4)	
	RR		18 (21.5)	18 (16.2)	

^a^Results are expressed as mean ± SD (range). NRs, non-responders; SVRs, sustained virological responders; RRs, responder–relapsers.

^b^ statistical assessment of each variable in NRs and SVRs in the total group.

A selective analysis was performed in the total group of 111 patients. The aim was to study the expression of 6 miRNAs (mir-122, mir-196b, mir-296-5p, mir-448, mir-431 and mir-218) and 30 mRNAs (S1 Table). While the 6 miRNAs were previously associated with HCV infection [17,18], the 30 selected genes were mainly involved in IFN signalling, cell adhesion and cell junction or encoding growth factors (S1 Table) [26,27]. Using liver biopsies, the fibrosis was staged according to the METAVIR scoring system: F0, no fibrosis; F1, portal fibrosis without septa; F2, portal fibrosis with rare septa; F3, numerous septa without cirrhosis; F4, cirrhosis [28].

### Histologically normal controls

Percutaneous liver biopsy specimens were taken from 9 adults with mild elevation of alanine aminotransferase (ALT) with no cause of liver disease (medication, alcohol, chronic viral hepatitis, autoimmune processes and metabolic disease) [29]. All the specimens were histologically normal with absence of inflammation, fibrosis, pathological pattern and less than 5% steatosis.

### IFNL3 genotyping

The patients were genotyped for rs12979860 using direct sequencing (Applied Biosystems), as previously described [10]. Primers used are available on request. Free circulating DNA was extracted from 500μl serum sample (Qiagen). The PCR products were separated on an ABI3130 sequencer, and analysed with SEQSCAPE 2.6 (Applied Biosystems).

### Statistical analysis

As the miRNA and mRNA levels did not fit a Gaussian distribution, (a) both the miRNA and mRNA levels in each subgroup of samples were characterized by their median values and ranges, rather than their mean values and coefficients of variation, and (b) relationships between the molecular markers and histological and viral parameters were tested using the nonparametric Mann–Whitney *U* test (Mann and Whitney, 1947). For miRNAs, differences between two populations were judged significant at confidence levels greater than 95% (*p* < 0.05) and a fold between the two populations more than 2 or a confidence level greater than 99% (p<0.01).

The selected miRNAs were assessed in the validation sample (excluding patients who were RR) using the same methodology. Deregulated miRNAs identified at this validation stage were candidate covariates for further analysis.

To identify independent predictors of SVR, we performed logistic regression modelling comparing NRs and SVRs in the total group (screen + validation, excluding RR patients). We performed univariate analyses to test the association between each candidate covariate and the response using the Wald test and we calculated the associated odds-ratios (OR). Covariates with a p-value <0.05 were selected for further multivariate analysis. In multivariate analysis a backward selection strategy was used to select independent predictors. The performance of the predictive selected model was assessed using the area under the receiver operating characteristic (ROC) curve. We used bootstrapping with 1,000 replicates of the total sample for internal validation of the predictive performance of the model as described in a previous study [30].

The material and methods of the RNA extraction and the RT-q-PCR are detailed in the S1 Methods.

## Results

### Patients

Baseline characteristics of the 111 patients studied are presented in Table 1. Patients are mainly male (n = 70 in the total group). The mean age at liver biopsy was 47.1 years. Genotypes were represented as follows with 51.2%, 10.8%, 13% and 23.2% of patients with respectively genotypes 1, 2, 3 and 4, in the total group of patients. Genotypes 1 and 4 are the most frequent in patients followed at Beaujon Hospital.

Patients from the screen group (n = 28) had mainly mild (F1) (n = 11) and moderate (F2) fibrosis (n = 16). In the screen group, patients with more advanced fibrosis were excluded to avoid identification of miRNAs that may be rather associated with advanced fibrosis than with response to PEG-IFN plus ribavirin. In the validation group, 23, 24, 17 and 17 patients were respectively F1, F2, F3 and F4. The fibrosis stage was unknown for 2 patients. Out of the 111 patients, 57 were SVR, 36 NR and 18 RR.

In the total group (excluding RR patients), Gamma-glutamyltransferase (per IU/L increase, OR = 0.991; 95% CI: 0.986–0.997; p = 0.0040), HCV viral load (per log10 cp/ml increase, OR = 0.311; 95% CI: 0.145–0.675; p = 0.0032) and HVC genotype 1 (vs. others, OR = 0.093; 95% CI: 0.020–0.444; p = 0.0029) were associated with lower rates of SVR. Moreover, *IFNL3* rs12979860 TT genotype was associated with NR (vs. CC or CT, OR = 0.271; 95%:CI 0.101–0.729; p = 0.0097).

### MiRNAs expression

#### Global miRNA approach.

**Fig 1:**
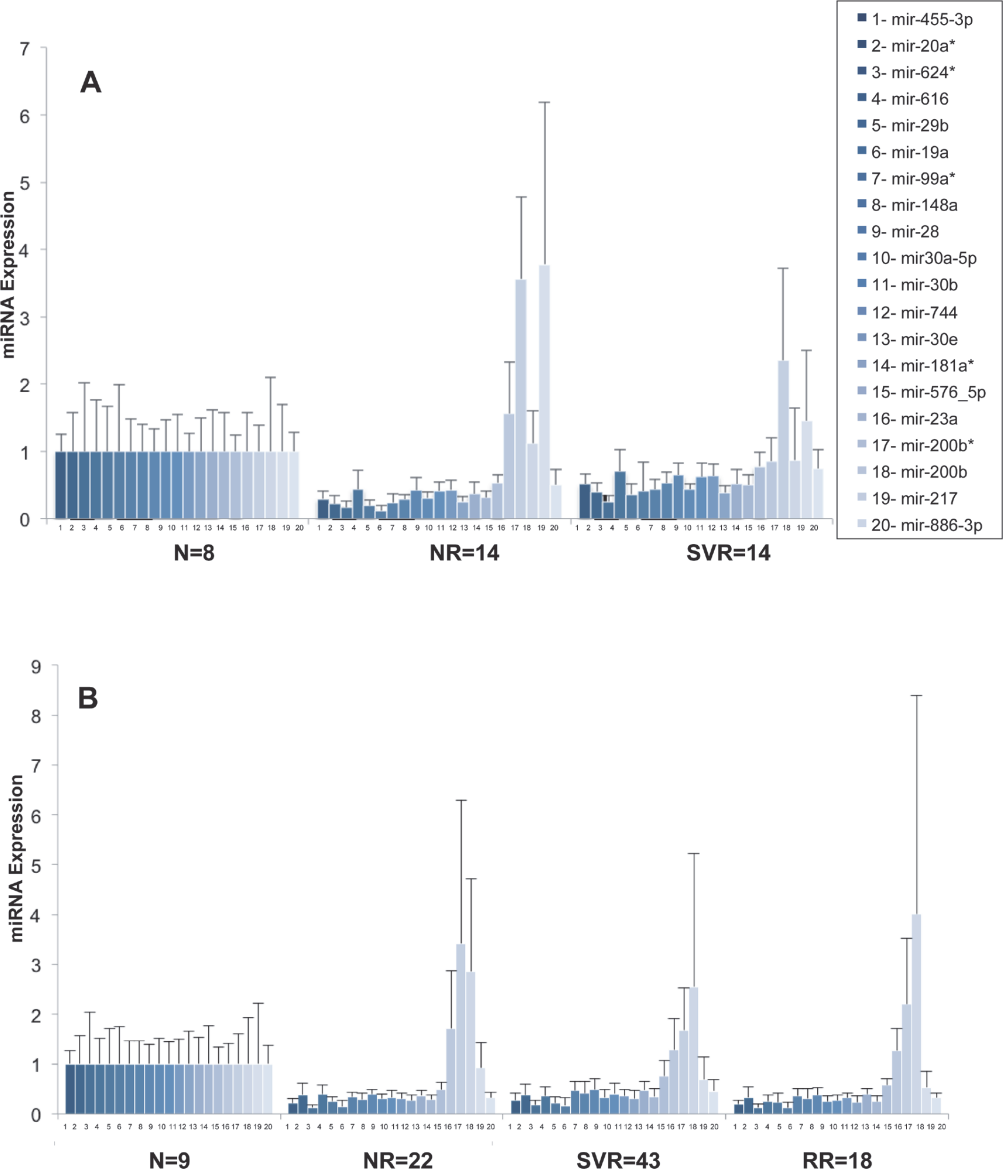
Modification of miRNAs expression in SVRs and NRs. Total RNAs was extracted and analyzed for miRNAs content by RT q-PCR. The ΔCt for each miRNA was calculated and normalized to the ΔCt value of SNORD44, in each biopsy. The histograms represent the mean expression of miRNAs/SNORD44 within the groups of patients NRs, SVRs and RRs normalized to the expression of the same miRNA within the group of normal patients. The association of miRNAs expression with viral response was calculated using the Wald test. 1A- Out of 851 miRNAs, 20 miRNAs were particularly deregulated between NRs and SVRs in the screen group (p<0.05 with at least a 2-fold difference between NRs and SVRs or p<0.01). 1B- The expression of the 20 miRNAs previously selected was assessed by RT q-PCR, in the validation group, independently. N = normal non-infected patients, NR = non-responders, RR = responders-relapsers, SVR = sustained virological responders.

Out of the 851 miRNAs studied, 20 were particularly deregulated (confidence levels greater than 95% (*p* < 0.05) and a fold between the two population more than 2 or a confidence level greater than 99% (p<0.01) between NRs and SVRs, in the screen group) (Fig. 1A and Table 2A). Four out of the 20 miRNAs selected showed the same deregulation of expression in the screen group and the validation group (Fig. 1B and Table 2B). Mir-99a, mir-181a* and mir-23a were down-regulated in NRs whereas mir-217 was accumulated in NRs compared to SVRs.

**Fig 2:**
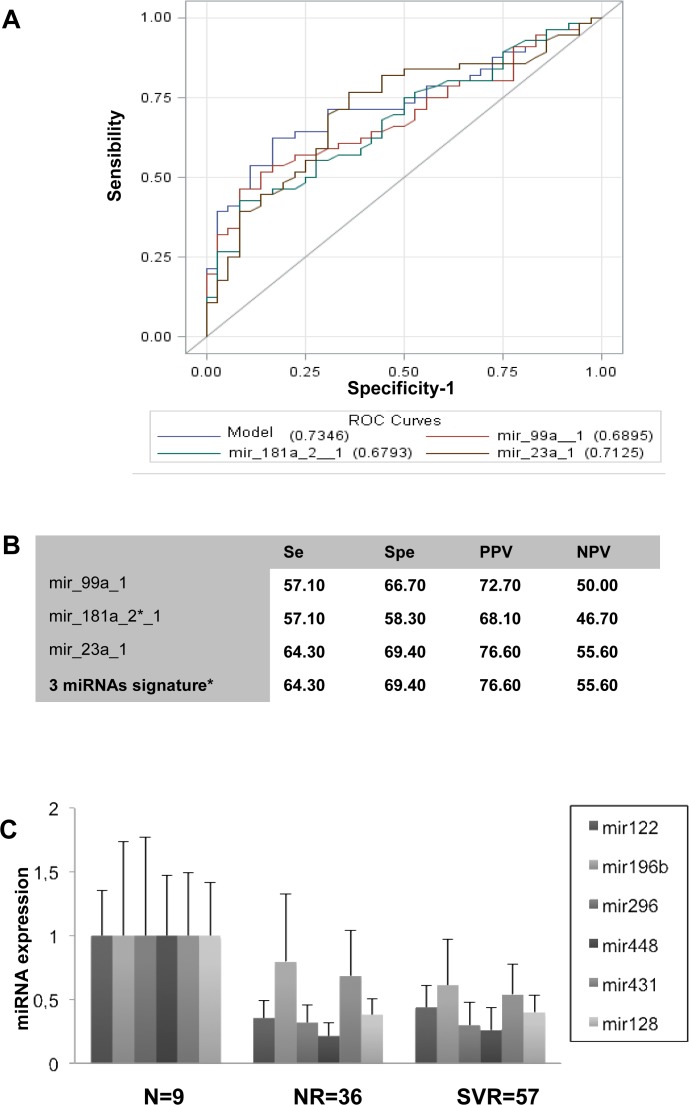
Identification of a 3 miRNAs signature (mir-99a, mir-23a and mir-181a*) predictor of SVR and selective analysis of miRNAs expression in NRs and SVRs. 2A- The receiver Operator Curves (ROC) were calculated for each miRNAs and for the signature based on the combination of the 3 miRNAs. 2B- The sensibility (Se), specificity (Spe), positive predictive value (PPV) and negative predictive value (NPV) were calculated for each miRNA isolated and for the combination of the 3 miRNAs. 2C- The expression of mir-122, mir-196b, mir-296-5p, mir-448, mir-431 and mir-128 was analyzed by RT q-PCR in the total group of patients (n = 111). The ΔCp (ΔCp_*t*_ = 2^*ΔCpsample*^) for each miRNAs was calculated and normalized to the ΔCp value of SNORD44 in each biopsy. The histograms represent the mean expression of miRNA/SNORD44 within the groups of patients NRs, SVRs and RRs normalized to the expression of the same miRNA within the group of normal patients. The association of mRNAs expression with viral response was calculated using the Wald test. N = normal un-infected patients, NR = non-responders, RR = responders-relapsers, SVR = sustained virological responders.

The OR were calculated for each of the 4 miRNAs, 3 were statistically significant (mir-99a, mir-181a* and mir-23a) (Fig. 2). The AUC of the model consisting in the combination of mir-99a, mir-181a* and mir-23a was 0.7346 (Fig. 2A). The sensibility, specificity, positive predictive value and negative predictive value of each miRNAs and the model combining the 3 miRNAs are represented on Fig. 2B.

**Table 2:** Differentially expressed miRNAs between NRs, SVRs and RR, prior to treatment.

	Screen			Validation			
miRNAS	NR	SVR	pvalue	NR	SVR	RR	pvalue
mir-455-3p	0.211	0.498	0.002	0.168	0.213	0.171	0.44
mir-20a*	0.202	0.415	0.009	0.343	0.406	0.355	0.81
mir-624*	0.330	0.672	0.006	0.357	0.410	0.269	0.16
mir-616	0.450	0.904	0.044	0.374	0.351	0.246	0.39
mir-29b	0.244	0.482	0.011	0.356	0.276	0.211	0.30
mir-19a	0.155	0.301	0.025	0.092	0.101	0.057	0.80
**mir-99a**	**0.223**	**0.412**	**0.002**	**0.343**	**0.478**	**0.295**	**0.0382**
mir-148a	0.238	0.424	0.000	0.258	0.361	0.224	0.19
mir-28	0.344	0.581	0.004	0.411	0.497	0.389	0.31
mir30a-5p	0.317	0.487	0.005	0.375	0.352	0.259	0.98
mir-30b	0.568	0.870	0.011	0.312	0.412	0.244	0.53
mir-744	0.449	0.676	0.008	0.422	0.534	0.448	0.30
mir-30e	0.251	0.371	0.004	0.367	0.374	0.282	0.91
mir-181a*	**0.400**	**0.591**	**0.005**	**0.522**	**0.622**	**0.486**	**0.0423**
mir-576_5p	0.568	0.772	0.008	0.600	0.697	0.510	0.28
**mir-23a**	**0.602**	**0.763**	**0.008**	**0.558**	**0.804**	**0.689**	**0.009**
mir-200b*	1.491	0.891	0.005	1.385	1.424	1.619	0.51
mir-200b	3.985	1.986	0.024	2.471	2.035	2.604	0.23
**mir-217**	**2.043**	**0.770**	**0.021**	**5.932**	**2.042**	**2.585**	**0.031**
mir-886-3p	6.899	2.474	0.004	2.081	1.025	0.923	0.1222

#### Candidate miRNAs approach.

In our study, mir-122 was neither significantly decreased in NRs compared to SVRs in the screen group (p = 0.077) nor in the total group (p = 0.06) (Fig. 2C). None of the miRNAs regulating HCV replication in vitro (mir-196b p = 0.499, mir-296-5p p = 0.253, mir-431 p = 0.128, mir-448 p = 0.971) showed difference of expression prior to treatment, in NRs and SVRs (Fig. 2C). The expression of mir-296-5p (p = 0.002), mir-122 (p<0.001), mir-448 (p<0.001) and mir-128 (p<0.001) was significantly down-regulated in patients with chronic hepatitis C compared to normal non-infected patients (Fig. 2C).

**Fig 3:**
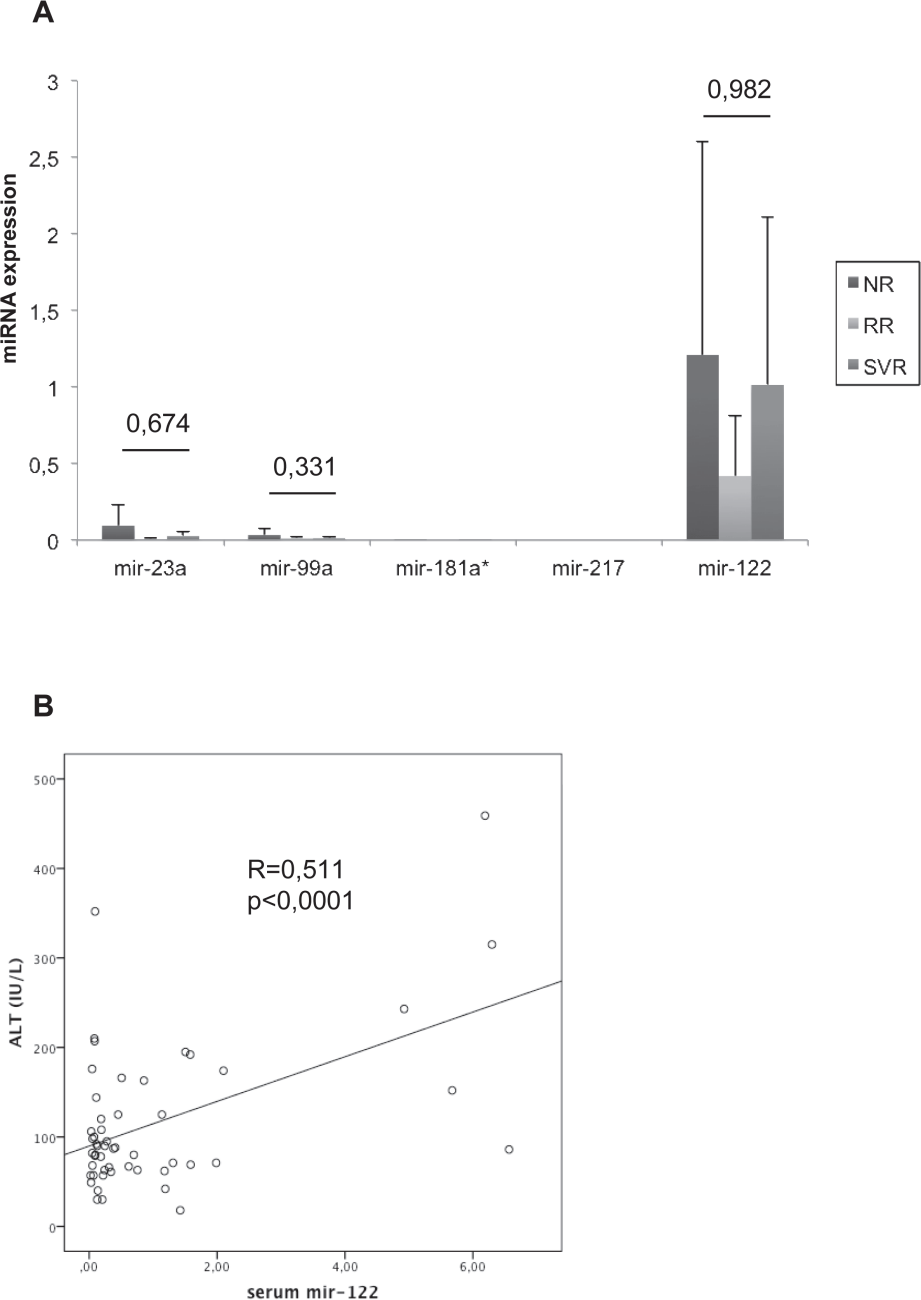
Modification of miRNAs expression in the serum of patients with chronic hepatitis C. 3A- Mir-23a, mir-99a, mir-181a*, mir-217 and mir-122 were detected by RT-q-PCR in 68 serums (NR = 26, RR = 10, RR = 32). The ΔCt for each miRNAs were calculated and normalized to the ΔCt value of *c*.*elegans* mir-39. The histograms represent the mean expression of miRNAs/mir-39 within the groups of patients NRs, RRs and SVRs. 3B- Correlation between mir-122 and alanine amino transferase, in the serum. The correlation was assessed using the Spearman correlation test. NR = non-responders, RR = responders-relapsers, SVR = sustained virological responders.

The expression of mir-23a, mir-99a, mir-181a*, mir-217 and mir-122 was investigated, in 68 serum samples available (NR = 26, RR = 10, RR = 32) (Fig. 3). The level of expression of mir-23a and mir-99a were low compared to mir-122 (Fig. 3A). None of mir-23a, mir-99a and mir-122 was differentially expressed between SVRs and NRs (Fig. 3A). Interestingly, only serum mir-122 and alanine amino transferase were strongly correlated (Fig. 3B), as we previously described [23]. Mir-217 and mir-181a* were not detected efficiently in our serum samples (Fig. 3). Moreover, the expression of mir-23a, mir-99a and mir-122 were not correlated in serum and liver samples (Table 3).

**Table 3:** Correlation between hepatic and serum expression of miRNAs.

mir-23a	R Pearson	-0.197
	pvalue	0.325
mir-99a	R Pearson	-0.370
	pvalue	0.090
mir-181a*	R Pearson	0.293
	p value	0.573
mir-217	R Pearson	NA*
	p value	NA*
mir-122	R Pearson	0.038
	p value	0.781

*NA: not applicable.

### Modification of mRNAs expression in NRs and SVRs

**Fig 4:**
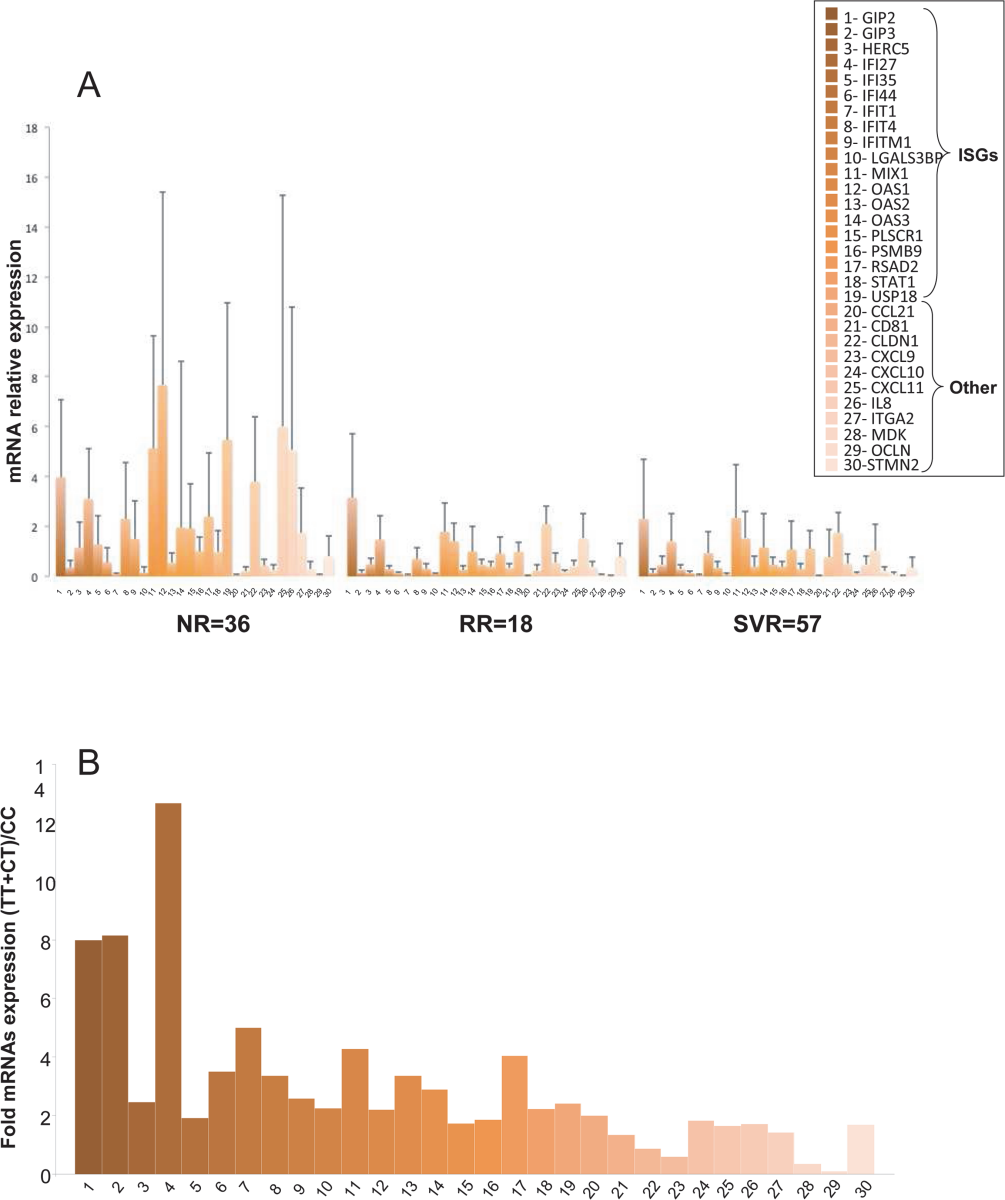
Differential mRNAs expression in NRs and SVRs prior to treatment. 4A- The hepatic expression of 25 mRNAs was assessed in the total group of patients by RT q-PCR. The ΔCp (ΔCp_*t*_ = 2^*ΔCpsample*^) value was calculated for each mRNA expression and normalized to the ΔCp value of RPLP0 in each liver biopsy. The histograms represent the mean value of the expression of mRNA in NRs, SVRs and RRs. The expression values were compared by non-parametric test. 4B- High expression of ISGs is associated with IFNL3 TT genotype. The intra-hepatic expression of 25 mRNAs was analyzed by RT-qPCR from total RNAs extracts. IFNL3 rs12979860 polymorphism was determined by direct sequencing. The ΔCp (ΔCp_*t*_ = 2^*ΔCpsample*^) value was calculated for each mRNA expression and normalized to the ΔCp value of RPLP0 in each liver biopsy. The histograms represent the mean value of the ratio of each mRNA expression in TT + CT /CC patients. ISGs: Interferon stimulated genes non- ISGs: other type of genes. NR = non-responders, RR = responders-relapsers, SVR = sustained virological responders.

From the 30 mRNAs tested 25 were deregulated between NRs and SVRs (Fig. 4A and Table 4). Interestingly all the ISGs assessed were accumulated in NRs compared to SVRs (Fig. 4A and Table 4). The ORs were calculated for each mRNAs (Table 4).

ISGs were particularly over-expressed in rs12979860 TT (16.125 to 2.07 fold Fig. 4B and S2A and S2B Tables) patients compared to CC (Fig. 4B). The 3 co-receptors of HCV entry occludin (*OCLN)* CD81 molecule *(CD81)* and claudin 1 (*CLDN1)* were only accumulated in NRs compared to SVRs and not in *IFNL3* TT compared to CC patients (Table 4 and S2 Table).

**Table 4:** Differentially expressed mRNAs between NRs and SVRs in patients with CHC prior to treatment.

**mRNAs**	**OR** ^a^	**95 % CI** ^b^	pvalue
**1-GIP2**	0.818	0.711–0.940	0.0047
**2-GIP3**	0.035	0.003–0.358	0.0048
**3-HERC5**	0.300	0.130–0.695	0.0050
**4-IFI27**	0.612	0.470–0.798	0.0003
**5-IFI35**	0.069	0.013–0.361	0.0015
**6-IFI44**	0.048	0.005–0.466	0.0088
**7-IFIT1**	<0.001	<0.001–0.004	0.0028
8-IFIT4	0.745	0.544–1.019	0.0653
**9-IFITM1**	0.266	0.100–0.711	0.0083
**10-LGALS3BP**	0.004	<0.001–0.172	0.0039
**11-MIX1**	0.843	0.742–0.957	0.0082
**12-OAS1**	0.656	0.492–0.875	0.0041
13-OAS2	0.591	0.268–1.306	0.1934
**14-OAS3**	0.765	0.626–0.934	0.0086
**15-PLSCR1**	0.327	0.147–0.729	0.0062
**16-PSMB9**	0.046	0.010–0.213	<.0001
**17-RSAD2**	0.761	0.590–0.983	0.0368
**18-STAT1**	0.139	0.036–0.537	0.0042
**19-USP18**	0.581	0.397–0.849	0.0051
**20-CCL21**	<0.001	<0.001 - <0.001	0.0032
21-CD81	1.282	0.792–2.076	0.3125
**22-CLDN1**	0.523	0.360–0.760	0.0007
23-CXCL9	1.133	0.425–3.017	0.8027
**24-CXCL10**	0.014	<0.001–0.324	0.0077
**25-CXCL11**	0.371	0.183–0.751	0.0059
**26-IL8**	0.711	0.514–0.982	0.0382
**27-ITGA2**	0.159	0.045–0.565	0.0045
**28-MDK**	0.005	<0.001–0.159	0.0030
**29-OCLN**	<0.001	<0.001 - <0.001	0.0070
30-STMN2	0.935	0.777–1.124	0.4730

^a^ Odds ratio;

^b^ 95% Confidence Interval

### Design of a signature to predict SVR

**Fig 5:**
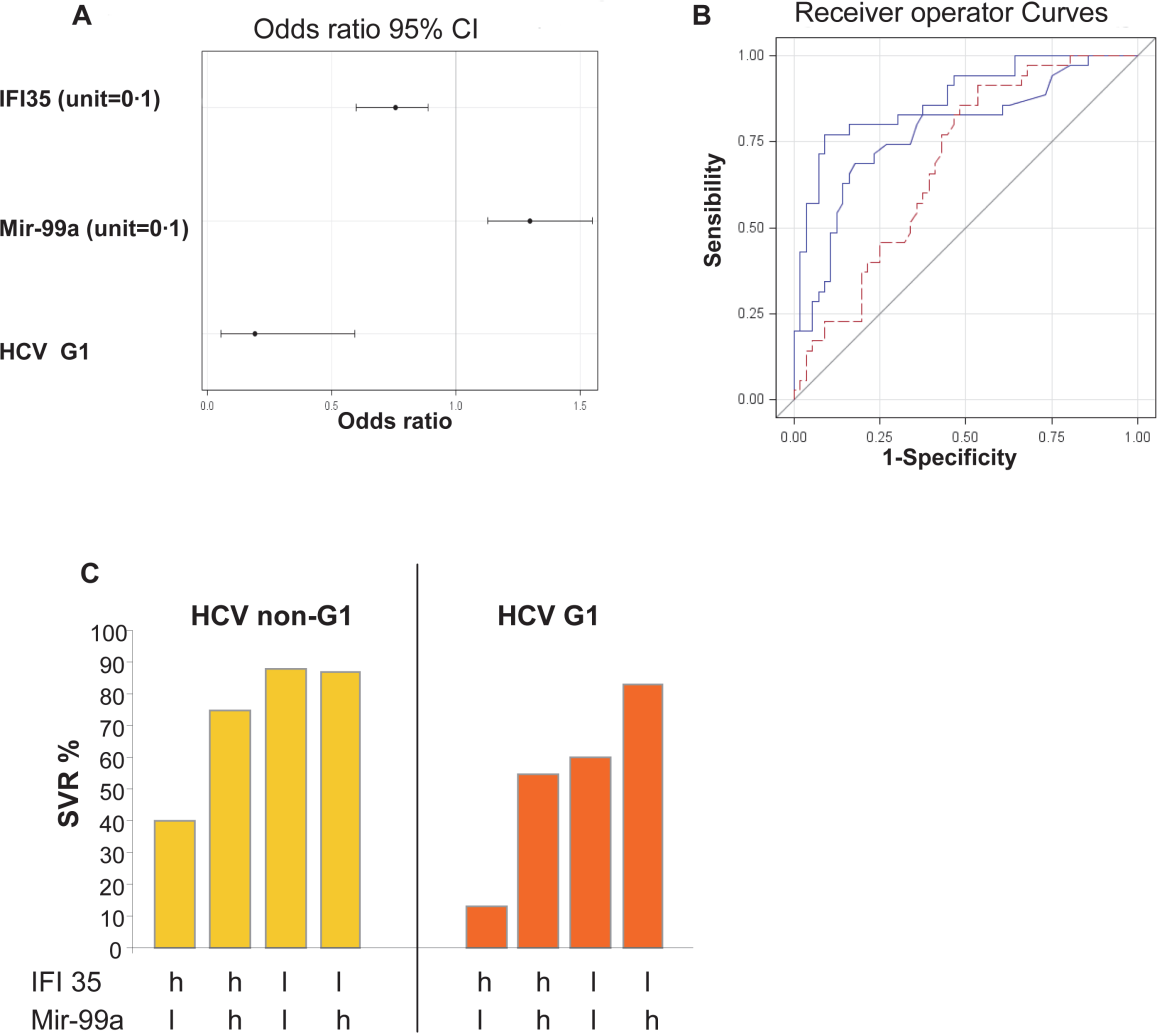
Identification of a signature predicting SVR. Clinical, miRNAs and mRNAs expression data were analyzed by multivariate analysis. 5A- ORs are presented for the 3 variables of the signature: mir-99a, IFI35 and HCV genotype 1. 5B- Receiver Operator Curves for prediction of viral response of the signature (AUC 0.876) (genotype 1, IFI35 and mir-99a expression) based on IFI35 expression (AUC 0.777) and mir-99a (AUC 0.688). 5C- Schematic representation of the prediction of SVR. Patients with genotype non-1 had high SVR rates when they express low level of IFI35 (l for low, < mean IFI35 expression). HCV genotype 1 infected patients with low level of IFI35 and high level of mir-99a (h for high, > mean mir-99a expression) showed high SVR rates.

The clinical and biological data, *IFNL3* polymorphism, mRNAs and miRNAs expression were analyzed by multivariate analysis. *IFNL3* polymorphism was not independent of ISGs expression (S3 Table). ISGs were particularly over-expressed in NRs compared to SVRs. It is likely that ISGs expression is a stronger predictor than *IFNL3* SNPs, considering the results of multivariate analysis. Mir-99a (per unit of relative mir-99a expression increase OR = 6.053 95% CI: 1.897–19.314 p = 0.0024) *IFI35* (per unit of relative *IFI35* expression increase OR = 0.069 95% CI:0.013–0.361 p = 0.0015) and HCV genotype 1 (vs. other genotypes OR = 0.093 95% CI: 0.020–0.444 p = 0.0029) were combined in a model predictor of SVR (Fig. 5A). The details about the selection of the variables in the multivariate analysis are presented in S3 Table. Overall the variables included in the multivariate analysis only mir-99a, *IFI35* and HCV genotype 1 were independent predictors of SVR (p = 0.0051 p = 0.0011 and p = 0.0059 respectively Wald test) (Fig. 5B). Interestingly the model predicts SVR with a positive predictive value of 86.54% (and a negative predictive value of 71.8%), a sensibility of 80% and a specificity of 80.4%. In patients infected with genotype 1 both IFI35 and mir-99a expression were important to predict SVR. However, in patients infected with either genotype 2, 3, 4 or 5, *IFI35* expression alone was a predictor of SVR (Fig. 5C).

For the identification of mir-99a, in the screen group, we use of a validation procedure on an independent sample prevented inflation of type I error and false discovery rate. A simple calculation under the hypothesis that all 20 miRNAs detected in the screening stage were false positive findings (a conservative scenario) showed that the likelihood of 4 (or more) false positive results on the validation sample (using a type I error at 5%) was 1.6%. In other words, it was unlikely to observe 4 miRNAs detected on the validation stage if all miRNAs detected on the test stage were false positive. Further analysis showed that only mir-99a was an independent predictor of SVR and mir-99a was integrated in the signature to predict SVR, with a p-Value of 0.0051 in multivariate analysis. We are confident that this result is unlikely related to inflation of type I error. However, further analysis will be needed to confirm the role of mir-99a in the prediction of SVR.

## Discussion

### Identification of miRNAs associated with SVR

The major novelty of our work consists in the identification of 4 miRNAs (mir-23a, mir-99a, mir-181a*, and mir-217) differentially expressed between NRs and SVRs.

The expression of mir-23a, mir-99a and mir-181a* was increased in SVRs compared to NRs. However, mir-217 was reduced in SVRs compared to NRs. Previous reports described hepatic expression of mir-99a, mir-23a and mir-181a suggesting processing of the pre-Mir and co-expression of mir-181a* [31–33]. Interestingly, in malignant cells, whereas the expression of some miRNAs is increased, the widespread under-expression of miRNAs is a more common phenomenon [34]. The modification of miRNAs expression profile might suggest that in NRs, hepatic cells may be more likely to undergo malignant process than in SVRs.

Interestingly, Mir-217 has been described as an oncogene [35] and a reduction of mir-99a expression has been reported in HCC tissues [31]. Moreover, restoration of mir-99a, *in vitro*, dramatically suppressed HCC cell growth by inducing G1 phase cell cycle arrest [31]. Mir-99a is increased during TGFB-induced epithelial to mesenchymal transition (EMT) [36]. Since EMT is involved during liver fibrogenesis, the expression of mir-99a may increase during fibrosis. Surprisingly, in our cohort, the expression of mir-99a is reduced in F2-F4 vs. F1 (p = 0.028) and in F3-F4 vs. F1F2 (p = 0.035) (S1 Fig). The EMT, in the liver may not induce a reduction of mir-99a expression. The reduction of mir-99a in NRs and at later stages of fibrosis may indicate that these two groups of patients have a higher risk to develop HCC.

The over-expression of mir-23a has been described in mouse models developing non-alcoholic steato-hepatitis, and HCC [32]. Moreover, mir-23a down-regulates interleukin-6 receptor [37] whereas high level of circulating IL-6 has been associated with NR [38]. The modulation of mir23a expression may regulate IL6 signalling in patients with CHC.

Interestingly, hepatic mir-181a* was up regulated in patients with SVR compared to NRs after PEG-IFN/RBV therapy [39]. This result supports our data and strongly suggest an up-regulation of mir-181a* expression in patients with SVRs.

Only few putative mRNAs targets of mir-23a, mir-217, mir-99a and mir-181a* have been described, so far. A deeper analysis of these miRNAs activity may help to elucidate the mechanisms of NR to IFN-based therapy.

A previous study investigating the modification of miRNAs expression, didn’t report the same set of miRNAs associated with SVR [16]. However, the authors didn’t validate their results using an independent group of patients and included only patients with Japanese ancestry [16]. These discrepancies may explain the identification of different miRNAs associated with SVR.

Mir-122 showed a trend of down-regulation in NRs. In a previous work, we reported a reduction of mir-122 in NRs plus RRs as compared to SVRs patients [23]. In agreement, in the current cohort, when RRs were added to the group of NRs, the difference of mir-122 expression between SVRs and NRs plus RRs was statistically significant (p = 0.02).

MiR-196, miR-296-5p, miR-431 and miR-448 are all induced by IFN α/β and contain putative recognition sites within HCV genome [17]. Whereas ISGs were significantly accumulated in NRs, no difference of expression of IFN-induced miRNAs was observed. Interestingly, this data raises the hypothesis that activation of miRNAs induced by IFN and ISGs, in NRs, may involve different signalling pathways.

In the serum, the level of expression of mir-23a and mir-99a were low compared to the one of mir-122 (Fig. 3A). Interestingly, serum mir-99a was down-regulated in patients with HCV compared to normal controls and patients with chronic hepatitis B [40]. This discrepancy may explain the low level of serum mir-99a detected in our cohort. Circulating mir-23a was suggested to be associated with type 2 diabetes [41]. However, in our cohort, none of the patients for whom serum was available, had diabetes. So we were unable to investigate this association. Interestingly, mir-122 was expressed in the serum and strongly correlates with Alanine amino transferase (Fig. 3B), confirming our previous results [23]. The levels of expression of mir-217 and mir-181a* were too low to be detected efficiently (Fig. 3A). To our knowledge, there are no study that reported efficient detection of serum mir-217 and mir-181a* in human serum samples.

### Analysis of mRNAs associated with response

All the ISGs studied were over-expressed in NRs, prior to treatment (Fig. 4A and Table 4). IFI35 is involved in the antiviral functions of IFN against viral infections. The over-expression of IFI35 suppresses the activation of IFNβ and ISG56 promoters [42]. Interestingly, in patients with NR, the higher expression of IFI35 may limit the efficacy of PEG-IFN to activate the expression of ISGs.

An interesting additional finding was the association of ISGs expression with the unfavourable T allele of rs12979860 IFNL3 (Fig. 4B and S2A and S2B Tables), as it was previously described [43–45]. Several studies reported that intra-hepatic ISGs expression is a better predictor of SVR than *IFNL3* polymorphisms [44,45]. The expression of a set of 3 ISGs (*ISG15*, *IFI27 and IFI6*) has been associated with rs12979860 SNPs [46]. *IFNL3* polymorphisms have been suggested to be associated with a specific cell-type modulation of ISGs expression (*MxA*, *PKR*, *OAS1* and *ISG15*) in hepatic cells and PBMCs [43].

### Predictive signature of SVR

We identified a signature combining the hepatic expression 3 miRNAs (mir-23a, mir-99a and mir-181a*) to predict SVR (AUC: 0.7346) (Fig. 2A). In our study, the accumulation of intra-hepatic ISGs expression, prior to treatment was more highly associated with NR than with *IFNL3* rs12979860 TT genotype. The multivariate analysis of all clinical and biological data, miRNAs and mRNAs expression and *IFNL3* SNPs, allowed us to build a signature (IFI35, mir-99a and HCV genotype) which is associated with a high predictive positive value (86.54%), sensibility (80%) and specificity (80.4%). None of the miRNAs identified showed a correlation between its hepatic and its serum expression (Table 3). Therefore, the expression of mir-99a alone or both mir-23a, mir-99a and mir-181a* has to be assessed in the liver, to provide enough information to predict SVR.

In a multivariable regression model, studying a HCV genotype 1 cohort, *IFNL3* rs12979860 CC genotype was the strongest pre-treatment predictor of SVR (OR = 5.2; 95% CI: 4.1–6.7) with only a sensitivity of 56%, a specificity of 79%, a positive predictive value of 69% and a negative predictive value of 68% [47]. Accordingly, in our cohort, *IFNL3* alone showed only moderate accuracy to predict SVR. The predictive value of *IFNL3* polymorphism is stronger in HCV genotypes 1 and 4 than in genotypes 2 and 3 [10,48]. Therefore, in our cohort combining all genotypes, the accuracy of *IFNL3* may be reduced. The combination of mir-99a, *IFI35* expression and HCV genotype was a higher predictive factor, at baseline, than IFNL3 polymorphism (Fig. 5).

In the current cohort (111 patients) both the 3-miRNAs-signature (AUC = 0.74) and the multi-variable signature (AUC = 0.876) showed higher accuracy to predict SVR than the 2-ISGs-signature (*IFI27* and *CXCL9*) previously described by our group (AUC = 0.713) [6].

An independent study reported a 4-ISGs-signature (*IFI27*, *GIP3/ISG15*, *RSAD2* and *HTATIP2*) as stronger predictor of SVR than IFNL3 SNPs [44]. However, the authors only investigated the expression of 8 selected mRNAs in a single group of patients without any validation set [44].

Whereas major improvements have been achieved in the treatment of CHC with the development of DAAs, PEG-IFN and ribavirin remains an option for many patients worldwide. Therefore the prediction of responsiveness to PEG-IFN plus ribavirin remains an important issue. The assessment of the hepatic expression of mir-99a and IFI35 may help to discriminate patients with low chance to respond to PEG-IFN plus ribavirin. In clinical practice, performing a liver biopsy remains useful to stage fibrosis [49], since urgent initiation of treatment is recommended for patients with advanced fibrosis or compensated cirrhosis. Performing different tests (stage fibrosis and predict treatment response) on the same liver biopsy could optimize the use of this invasive procedure. Patients with low chance to respond to PEG-IFN plus ribavirin may be candidates for IFN-free regimen.

## Supporting Information

S1 Fig Down-regulation of hepatic mir-99a in patients with advanced fibrosis. Total RNAs was extracted and analyzed for mir-99a content by RT q-PCR. The ΔCt for mir-99a was calculated and normalized to the ΔCt value of SNORD44 in each biopsy. The histograms represent the mean expression of mir-99a/SNORD44 within the groups of patients with the different stages of fibrosis according to metavir score (F1 to F4), normalized to mir-99a within the group of normal patients. S1A- The level of expression of mir-99a was compared between patients with mild fibrosis (F1, n = 35) and those with moderate fibrosis to cirrhosis (F2-F4, n = 71). S1B- the level of expression of mir-99a was compared in patients with mild ad moderate fibrosis (F1-F2, n = 71) and those with with advanced fibrosis and cirrhoris (F3-F4, n = 35).
(TIF)

S1 Methods RNA extraction and RT-q-PCR detailed methods. The detailed methods for the RNA extraction and the RT-q-PCR methods are described in the S1 Methods.
(DOC)

S1 Table List of the genes selected for the analysis. (DOC)

S2 Table Fold of expression of selected genes between IFNL3 CC and TT patients. Interferon stimulated genes (ISGs) in 2A and non- ISGs in 2B.
(DOC)

S3 Table Description of the multivariate analysis. The analyses were run separately for miRNAs, baseline characteristics and mRNAs. Multivariate findings were combined in a final multivariate model with backward selection to predict SVR in 93 patients (57 SVRs and 36 NRs).
(DOCX)
