# Supplementary figures and images for: IFI35, mir-99a and HCV Genotype to Predict Sustained Virological Response to Pegylated-Interferon Plus Ribavirin in Chronic Hepatitis C
Source: PLoS One. 2015 Apr 6;10(4):e0121395. doi: 10.1371/journal.pone.0121395 (PMC4386819; doi:10.1371/journal.pone.0121395)

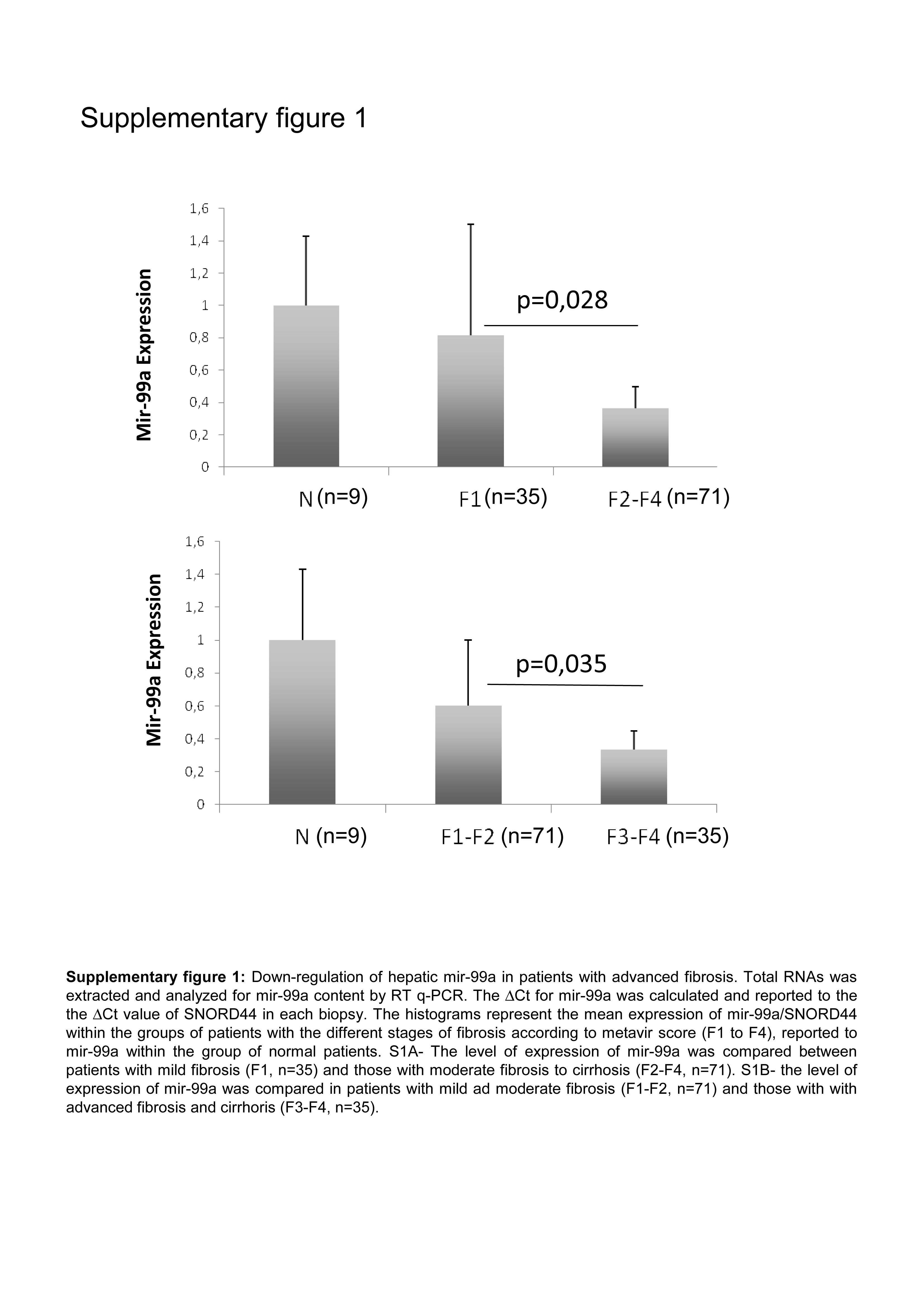

Supplement: S1 Fig — Total RNAs was extracted and analyzed for mir-99a content by RT q-PCR. The ΔCt for mir-99a was calculated and normalized to the ΔCt value of SNORD44 in each biopsy. The histograms represent the mean expression of mir-99a/SNORD44 within the groups of patients with the different stages of fibrosis according to metavir score (F1 to F4), normalized to mir-99a within the group of normal patients. S1A- The level of expression of mir-99a was compared between patients with mild fibrosis (F1, n = 35) and those with moderate fibrosis to cirrhosis (F2-F4, n = 71). S1B- the level of expression of mir-99a was compared in patients with mild ad moderate fibrosis (F1-F2, n = 71) and those with with advanced fibrosis and cirrhoris (F3-F4, n = 35). (TIF) [file pone.0121395.s001.tif]
