# Supplementary material for: IFI35, mir-99a and HCV Genotype to Predict Sustained Virological Response to Pegylated-Interferon Plus Ribavirin in Chronic Hepatitis C
Source: PLoS One. 2015 Apr 6;10(4):e0121395. doi: 10.1371/journal.pone.0121395 (PMC4386819; doi:10.1371/journal.pone.0121395)
Supplement: S1 Methods — The detailed methods for the RNA extraction and the RT-q-PCR methods are described in the S1 Methods. (DOC) [file pone.0121395.s002.doc]

**S1 Methods.** RNA extraction and RT-q-PCR detailed methods.

**RNA extraction**

Frozen liver biopsies were crushed and diluted in 1ml of iced RNAble (Eurobio). 100µl of chloroform was added to the mix. The upper phase was collected after centrifugation and the same volume of isopropanol was added. RNA was precipitated, by centrifugation, and washed in 80% Et-OH. The RNA pellet was resuspended in 20µl of RNAse and DNAse free deionised water. Total RNAs were extracted from 400µl of serum (Mirvana, Life science). Since there is no miRNA stably expressed in the serum, we added 5.6x10^8^ copies of mir-39 (*Caenorhabdditis elegans*, Qiagen) to each serum sample before the extraction. Mir-122 expression was then normalized to mir-39 expression values. Quantity and quality of total RNA were evaluated by measuring OD at 260nm.

**Real-time -qPCR**

Both mRNAs and miRNAs content were detected by real-time quantitative PCR (RT-qPCR), SYBR green. The PCR reactions were performed using the Light Cycler® 480 Real-Time PCR System. The theoretical and practical aspects of RT-qPCR using the light cycler LC480 (Roche) have been already described in details .

One ng of cDNA from either miRNAs or mRNAs reverse transcription reactions was added to each reaction. The amount of total mRNAs and miRNAs, in liver samples, were respectively normalized to the amount of endogenous RNA control gene RPLP0 (also known as 36B4; NM_001002) and SNORD44. A total of 7 non-coding RNAs (mir-25_1, SNORD44, mir-191_1, mir-103_1, SNORD48, RNU6B_2 and RNU5A_1) were analyzed in patients from the screen and normal groups. The expression of each these 7 non coding RNAs were compared and normalized to each other to identify the most accurate candidate to normalize the results. When the values were normalized to SNORD44, the expression of the other non-coding RNAs was the most stable between the 2 groups of patients. Therefore, SNORD44 was chosen as reporter non-coding RNAs for the rest of the experiments. For serum samples, the level of expression of each miRNA was detected and normalized to the amount of *c.elegans* mir-39.

Quantitative values are obtained from the crossing point (Cp), number at which the increase in the signal associated with exponential growth of PCR products begins to be detected using Light Cycler® 480 System Software (Roche Diagnostics, Mannheim, Germany), according to the manufacturer’s manuals.

Results of each mRNA and miRNA, are expressed as the N-fold differences in target gene expression relative to the RPLP0 gene, SNORD44 or *c.elegans* mir-39, and termed N*_target_*. This value was determined as N*_target_*= 2*^ΔCpsample^*, where the ΔCp value of the sample was determined by subtracting the average Cp value of the target gene from the average Cp value of the RPLP0 gene, SNORD44 or *c.elegans* mir-39.

**Global miRNA approach**

The expression 851 miRNAs were compared in NRs and SVRs from the screen group, using the pre-designed ready-to-use Human miscript Assay V (Qiagen). Briefly, all the miRNAs were elongated during reverse transcription and prior to RT-q-PCR. The generic region added to all reverse transcripts miRNAs consists in a polyA tail and a specific sequence of 20 bases designed by the manufacturer (Qiagen). This sequence is used as recognition site for a universal primer. The second primer targets specifically each miRNA and allows the specific amplification. The miRNAs deregulated between NRs and SVRs, within the screen group, were assessed individually and in an independent set of experiments in the validation group.

**Candidate miRNA approach**

We identified 6 miRNAs of particular interest, described in HCV infection and reviewed in . Mir-122 has been reported to play an important role in HCV replication . Mir-196, mir-296, mir-431, mir-448 and mir-128 were all induced by IFN α/β and had putative recognition site within HCV genome . The expression of these 6 miRNAs was analyzed by RT-q-PCR as described above.

**Selection of genes of interest**

The expression of 30 mRNAs was assessed by RT-q-PCR. The 30 genes were selected according to the literature (Supplementary table 1) . They belong mainly to ISG family, cell adhesion and cell junction or encode growth factor pathways.

**References:**

1. Bieche I, Asselah T, Laurendeau I, Vidaud D, Degot C, Paradis V, et al. Molecular profiling of early stage liver fibrosis in patients with chronic hepatitis C virus infection. Virology. 2005; 332: 130-144.

2. Asselah T, Bieche I, Laurendeau I, Paradis V, Vidaud D, Degott C, et al. Liver gene expression signature of mild fibrosis in patients with chronic hepatitis C. Gastroenterology. 2005; 129: 2064-2075.

3. Jopling CL, Yi M, Lancaster AM, Lemon SM, Sarnow P. Modulation of hepatitis C virus RNA abundance by a liver-specific MicroRNA. Science. 2005; 309: 1577-1581.

4. Pedersen IM, Cheng G, Wieland S, Volinia S, Croce CM, Chisari FV, et al. Interferon modulation of cellular microRNAs as an antiviral mechanism. Nature. 2007; 449: 919-922.

5. Estrabaud E, Vidaud M, Marcellin P, Asselah T. Genomics and HCV infection: progression of fibrosis and treatment response. J Hepatol. 2012;

6. Sarasin-Filipowicz M, Oakeley EJ, Duong FH, Christen V, Terracciano L, Filipowicz W, et al. Interferon signaling and treatment outcome in chronic hepatitis C. Proc Natl Acad Sci U S A. 2008; 105: 7034-7039.

7. Asselah T, Bieche I, Narguet S, Sabbagh A, Laurendeau I, Ripault MP, et al. Liver gene expression signature to predict response to pegylated interferon plus ribavirin combination therapy in patients with chronic hepatitis C. Gut. 2008; 57: 516-524.

8. Asselah T, Bieche I, Sabbagh A, Bedossa P, Moreau R, Valla D, et al. Gene expression and hepatitis C virus infection. Gut. 2009; 58: 846-858.
