# Supplementary material for: IFI35, mir-99a and HCV Genotype to Predict Sustained Virological Response to Pegylated-Interferon Plus Ribavirin in Chronic Hepatitis C
Source: PLoS One. 2015 Apr 6;10(4):e0121395. doi: 10.1371/journal.pone.0121395 (PMC4386819; doi:10.1371/journal.pone.0121395)
Supplement: S1 Table — (DOC) [file pone.0121395.s003.doc]

**S1 Table : List of the genes selected for the analysis.**

| **Function** | **Gene** | **Protein** | **Genbank accession** |
| --- | --- | --- | --- |
| **Cell adhesion and cell junction** | CLDN1 | Claudin 1 | NM_021101 |
|  | ITGA2 | Integrin a 2 | NM_002203 |
|  | OCLN | Occludin (virus entry) | NM_002538 |
|  |  |  |  |
| **Growth factors** | CCL21 | Chemokine (C-C motif) ligand 21 | NM_002989 |
|  | CXCL10 | Chemokine (CXC motif) ligand 10 | NM_001565 |
|  | CXCL11 | Chemokine (CXC motif) ligand 11 | NM_005409 |
|  | MDK | Midkine | NM_002391 |
|  | IL8 | Interleukin 8 | NM_000584 |
| **Interferon signaling pathway** |  |  |  |
|  | STAT1 | Signal transducer and activator of transcription 1 | NM_007315 |
| **Interferon inducible proteins** |  |  |  |
|  | HERC5 | Hect domain RLD5 | NM_016323 |
|  | GIP3/IFI6 | Interferon alpha inducible protein 6 | NM_022873 |
|  | IFI27 | Interferon alpha inducible protein 27 | NM_005532 |
|  | IFI35 | Interferon alpha inducible protein 35 | NM_005533 |
|  | IFI44 | Interferon induced protein 44 | NM_006417 |
|  | IFIT1 | Interferon induced protein with tetratricopeptide repeats 1 | NM_001548 |
|  | IFITM1 | Intereferon induced transmembrane protein 1 | NM_003641 |
|  | GIP2/ISG15 | Interferon alpha inducible protein (clone IFI-15K) | NM_005101 |
|  | MX1/IFI78 | Myxovirus (influenzae virus) resistance 1 | NM_002462 |
|  | LGALS3BP | Lectin, galactosidase-binding, soluble, 3 binding protein | NM_005567 |
|  | OAS1 | 2'-5' oligoadenylate synthetase 1 | NM_016816 |
|  | OAS3 | 2'-5' oligoadenylate synthetase 3 | NM_006187 |
|  | PLSCR1 | Phospholipid scramblase 1 | NM_021105 |
|  | PSMB9 | Proteasome subunit bta type 9 | NM_002800 |
|  | RSAD2 | Radical s-adenosyl methionin domain containing 2 | NM_080657 |
| **Various** | CD81 | CD81 molecule | NM_004356 |
|  | STMN2 | Stathmin-like 2 | NM_007029 |
|  | USP18 | Ubiquitin specific peptidase 18 | NM_017414 |
