# Supplementary material for: IFI35, mir-99a and HCV Genotype to Predict Sustained Virological Response to Pegylated-Interferon Plus Ribavirin in Chronic Hepatitis C
Source: PLoS One. 2015 Apr 6;10(4):e0121395. doi: 10.1371/journal.pone.0121395 (PMC4386819; doi:10.1371/journal.pone.0121395)
Supplement: S2 Table — Interferon stimulated genes (ISGs) in 2A and non- ISGs in 2B. (DOC) [file pone.0121395.s004.doc]

**S2 Table. Fold of expression of selected genes between IFNL3 CC and TT patients.** Interferon stimulated genes (ISGs) in 2A and non- ISGs in 2B.

2A

| **ISGs** | **Fold TT/CC^a^** | **pvalues** |
| --- | --- | --- |
| GIP2 | 12 | <0.0001 |
| GIP3 | 11.5 | <0.0001 |
| HERC5 | 3.05 | <0.0001 |
| IFI27 | 16.125 | <0.0001 |
| IFI35 | 2.73 | <0.0001 |
| IFI44 | 4.5 | <0.0001 |
| IFIT1 | 7 | <0.0001 |
| IFIT4 | 4.57 | <0.0001 |
| IFITM1 | 4.08 | <0.0001 |
| LGALS3BP | 3.17 | <0.0001 |
| MIX1 | 5.91 | <0.0001 |
| OAS1 | 3.57 | <0.0001 |
| OAS2 | 6.25 | <0.0001 |
| OAS3 | 4.83 | <0.0001 |
| PLSCR1 | 2.07 | 0.004 |
| PSMB9 | 2.48 | 0.001 |
| RSAD2 | 6 | <0.0001 |

2B

| **Non ISGs** | **Fold**  **TT/CC^a^** | **pvalues** |
| --- | --- | --- |
| CCL21 | 2 | 0.002 |
| CD81 | 1.33 | 0.332 |
| CLDN1 | 1.14 | 0.201 |
| CXCL9 | 0.592 |  |
| CXCL10 | 3 | 0.015 |
| CXCL11 | 2.34 | 0.105 |
| IL8 | 1.89 | 0.204 |
| ITGA2 | 1.21 | 0.351 |
| MDK | 8 | <0.0001 |
| OCLN | 1.5 | 0.055 |
| STAT1 | 2.64 | <0.0001 |
| STMN2 | 1.92 | 0.161 |
| USP18 | 3.57 | <0.0001 |
