# Supplementary material for: IFI35, mir-99a and HCV Genotype to Predict Sustained Virological Response to Pegylated-Interferon Plus Ribavirin in Chronic Hepatitis C
Source: PLoS One. 2015 Apr 6;10(4):e0121395. doi: 10.1371/journal.pone.0121395 (PMC4386819; doi:10.1371/journal.pone.0121395)
Supplement: S3 Table — The analyses were run separately for miRNAs, baseline characteristics and mRNAs. Multivariate findings were combined in a final multivariate model with backward selection to predict SVR in 93 patients (57 SVRs and 36 NRs). (DOCX) [file pone.0121395.s005.docx]

**S3 Table. Description of the multivariate analysis.**

The analyses were run separately for miRNAs, baseline characteristics and mRNAs. Multivariate findings were combined in a final multivariate model with backward selection to predict virological response in 93 patients (57 SVRs and 36 NRs).

**3A** mirRNAs: univariate and multivariate Odds-Ratios of Virological response

|  | OR univariate | 95%CI | P Value | OR multivariate (backward selection) | 95%CI | P Value |
| --- | --- | --- | --- | --- | --- | --- |
| Mir-99a (per unity) | 6.053 | 1.90-19.3 | 0.0024 | 6.053 | 1.90-19.3 | 0.0024 |
| Mir-181a-2 | 5.726 | 1.74-18.9 | 0.0041 |  |  | NS |
| Mir-23a | 8.911 | 2.16-36.8 | 0.0025 |  |  | NS |
| Mir-217 | 0.989 | 0.89-1.11 | 0.8520 |  |  | Not included |

Not significant at P<0.05 level

**3B** Baseline characteristics: univariate and multivariate Odds-Ratios of Virological response

|  | OR univariate | 95%CI | P Value | OR multivariate (backward sel) | 95%CI | P Value |
| --- | --- | --- | --- | --- | --- | --- |
| Geno1  Geno 4 | 0.093  0.226 | 0.02-0.44  0.04-1.24 | 0.0029  0.0881 | 0.208 | 0.06-0.71 | 0.0117  NS |
| Log CV | 0.311 | 0.14-0.68 | 0.0032 | 0.162 | 0.05-0.50 | 0.0017 |
| IL28 TT | 0.271 | 0.10-0.73 | 0.0097 | 0.163 | 0.04-0.63 | 0.0082 |
| GGT | 0.991 | 0.99-1.00 | 0.0040 |  |  | NS |

Not significant at P<0.05 level

**3C** mRNAs : Multivariate Odds-Ratios after backward selection.

(NB : univariate ORs are shown in table 3)

|  | OR multivariate  (backward selection) | 95%CI | P Value |
| --- | --- | --- | --- |
| IFI35 (per unity) | <0.001 | <0.001-0.073 | 0.0030 |
| IFITM1 | 91.145 | 2.73- >999 | 0.0117 |
| MDK | 0.003 | <0.001-0.225 | 0.0082 |

**3D** Final model: multivariate Odds-Ratios of Virological response

|  | OR multivariate  (backward selection) | 95%CI | P Value |
| --- | --- | --- | --- |
| Mir-99a (per unity) | 13.43 | 2.83-63.8 | 0.0011 |
| IFI35 (per unity) | 0.061 | 0.009-0.43 | 0.0051 |
| IFITM1 |  |  | NS |
| MDK |  |  | NS |
| Geno1 | 0.191 | 0.059-0.62 | 0.0059 |
| Log CV |  |  | NS |
| IL28 TT |  |  | NS |

**3F** Final model: multivariate Odds-Ratios of Virological response – with ORs expressed as per 0.1 unit for Mir-99a and IFI35 (as shown figure 5B).

|  | OR multivarié  (backward selection) | 95%CI | P Value |
| --- | --- | --- | --- |
| Mir-99a (per 0.1) | 1.297 | 1.11-1.52 | 0.0011 |
| IFI35 (per 0.1) | 0.756 | 0.62-0.92 | 0.0051 |
| IFITM1 |  |  | NS |
| MDK |  |  | NS |
| Geno1 | 0.191 | 0.059-0.62 | 0.0059 |
| Log CV |  |  | NS |
| IL28 TT |  |  | NS |
